# Experimental PCEP-Adjuvanted Swine Influenza H1N1 Vaccine Induced Strong Immune Responses but Did Not Protect Piglets against Heterologous H3N2 Virus Challenge

**DOI:** 10.3390/vaccines8020235

**Published:** 2020-05-18

**Authors:** Royford Bundi Magiri, Ken John Lai, George Kiremu Mutwiri, Heather Lynne Wilson

**Affiliations:** 1 Vaccinology & Immunotherapeutic Program, School of Public Health, University of Saskatchewan, Saskatoon, SK S7N 2Z4, Canada; royford.magiri@fnu.ac.fj (R.B.M.); ken.lai@usask.ca (K.J.L.); george.mutwiri@usask.ca (G.K.M.); 2 Vaccine & Infectious Disease Organization-International Vaccine Centre (VIDO-InterVac), University of Saskatchewan, 120 Veterinary Road, Saskatoon, SK S7N 5E3, Canada; 3 College of Agriculture, Fisheries and Forestry, Fiji National University, Suva 7222, Fiji

**Keywords:** polyphosphazene, adjuvant, pig, intradermal, influenza

## Abstract

Vaccination is the most efficient method of protection against influenza infections. However, the rapidly mutating viruses and development of new strains make it necessary to develop new influenza vaccines annually. Hence, vaccines that stimulate cross-protection against multiple influenza subtypes are highly sought. Recent evidence suggests that adjuvants such as PCEP that promote Th1-type T cell and Th2-type T cell immune responses and broad-spectrum immune responses may confer cross-protection against heterologous influenza strains. In this study, we evaluated whether the immunogenic and protective potential of PCEP-adjuvanted inactivated swine influenza virus H1N1 vaccine can protect pigs immunized against live H3N2 virus. Piglets were vaccinated via the intradermal route with PCEP-adjuvanted inactivated swine influenza virus (SIV) H1N1 vaccine, boosted at day 21 with the same vaccines then challenged with infectious SIV H3N2 virus at day 35 via the tracheobronchial route. The pigs showed significant anti-H1N1 SIV specific antibody titres and H1N1 SIV neutralizing antibody titres, and these serum titres remained after the challenge with the H3N2 virus. In contrast, vaccination with anti-H1N1 SIV did not trigger anti-H3N2 SIV antibody titres or neutralizing antibody titres and these titres remained low until pigs were challenged with H3N2 SIV. At necropsy (six days after challenge), we collected prescapular lymph nodes and tracheobronchial draining the vaccination sites and challenge site, respectively. ELISPOTs from lymph node cells restimulated ex vivo with inactivated SIV H1N1 showed significant production of IFN-γ in the tracheobronchial cells, but not the prescapular lymph nodes. In contrast, lymph node cells restimulated ex vivo with inactivated SIV H1N1 showed significantly higher IL-13 and IL-17A in the prescapular lymph nodes draining the vaccination sites relative to unchallenged animals. Lung lesion scores show that intradermal vaccination with H1N1 SIV plus PCEP did not prevent lesions when the animals were challenged with H3N2. These results confirm previous findings that PCEP is effective as a vaccine adjuvant in that it induces strong immune responses and protects against homologous swine influenza H1N1 virus, but the experimental H1N1 vaccine failed to cross-protect against heterologous H3N2 virus.

## 1. Introduction

Swine influenza virus (SIV) is a highly contagious acute respiratory disease of pigs [1]. There has been an increase in the genetic diversity of swine influenza A virus with the majority of the SIV infections in pigs are caused by subtypes H1N1, H1N2 and H3N2 with new reassortments such as H1N1pdm09 being identified [2,3,4,5]. Other subtypes such as H3N8, H4N8, H5N1 and H6N6 have been identified but they cause a minority of SIV infections. Swine influenza virus is endemic worldwide and it is responsible for significant economic losses to the swine industry each year [3]. In addition, SIV infections are a threat to public health since transmission from pigs to humans can occur, hence a vaccine that stimulates a rapid and long-lasting protective immune response to homologous and heterologous strains is highly sought. The most cost-effective public health tool available to control SIV infection is through effective vaccination. Current pig vaccines are comprised of inactivated H3N2 and H1N1virus [6]. Because subunit vaccines contain highly purified antigens, they are poorly immunogenic and require the addition of adjuvants to induce protective immune responses. Additionally, reformulation of the vaccine to include a potent adjuvant may improve the efficacy of existing SIV vaccines.

Adjuvants are routinely included in vaccines comprised of inactivated virus and/or subunit vaccines to augment the magnitude and quality of immune responses and by enhancing onset and extending duration of immunity. Many adjuvants can improve immune responses and promote protection against infection with homologous influenza virus strains in humans and animals. However, cross protection against heterologous virus strains remains a challenge in the development of influenza vaccines. A recent study by Clegg revealed that the combination adjuvant GLA-SE (a two-part adjuvant system containing glucopyranosyl lipid adjuvant (GLA), a formulated synthetic Toll-like receptor 4 agonist, and a stable emulsion (SE) of oil in water), but not the commercial SE adjuvant, conferred protection against heterosubtypic H5N1 challenge in mice and ferrets [7]. This cross-protection was apparently mediated via induction of Th1-mediated antibody responses [7]. Polyphosphazenes are high-molecular weight, water-soluble polymers that have been shown to promote and enhance long lasting immune responses with a variety of viral and bacterial antigens [8,9,10,11,12,13]. Poly (di (sodium carboxylatoethylphenoxy))-phosphazene (PCEP) promotes strong antigen-specific Th1- and Th2-type immune responses to influenza antigens in mice and pigs [10,13,14,15]. PCEP induced significant production of interleukin IL-1β, and IL-13 at the site of injection and IL-1β, and IL-6 at the draining lymph nodes. and we previously reported that pigs vaccinated via the intradermal route with PCEP-adjuvanted inactivated SIV H1N1 vaccine were protected against virulent challenge with homologous H1N1 [13]. PCEP injected intradermally into pigs in the absence of antigen induced significant production of interleukin IL-1β, and IL-13 at the site of injection and IL-1β, and IL-6 at the draining lymph nodes, possibly contributing to an immunocompetent environment [15]. In this study, we evaluated the immunogenicity and cross protective efficacy of PCEP-adjuvanted inactivated SIV H1N1 vaccine against virulent challenge with heterologous H3N2 in pigs.

## 2. Materials and Methods

### 2.1. Swine Influenza Virus Adsorption and Purification

In separate flasks, swine influenza virus H1N1 A/swine/Saskatchewan/18789/2002 (H1N1) and the challenge strain H3N2 (A/swine/Texas/4199-2/1998/H3N2 (Tx98)) isolates (swine influenza virus SK and Texas strain, respectively) were cultured in confluent Madin–Darby canine kidney (MDCK) cells at a multiplicity of infection (M.O.I) of 0.001 plaque-forming units (PFU)/mL. Cells were grown in tissue culture media MEM at 37 °C in 5% CO_2_ with nutation every 15 min. After 1 h, each inoculum was removed and 10 mL of DMEM with 0.2% heat-inactivated BSA (Sigma-Aldrich# A8806, Oakville, ON, Canada), 1 μg/mL of N-P-tosyl-phenylalanine chloromethyl ketone (TPCK) (MJS BioLynx, Brockville, ON, Canada) and 50 μg/mL of gentamicin were added per flask. The cultures were incubated in a humidified 5% CO_2_ atmosphere at 37 °C for 3 days or until 90% of cytopathic effect (CPE) was observed. Infected cell cultures were centrifuged at 200× *g* for 10 min at 14 °C. The supernatant was collected, aliquoted into small volumes, and stored at −70 °C until further purification was performed.

Virus containing supernatants were subjected to 112,700× *g* centrifugation for 2.5 h at 4 °C. The resulting pellet was collected in 500 μL TSE buffer (20 mM Tris, 2 mM EDTA & 150 mM NaCl, pH 7.4), placed upon a 30%/60% discontinuous sucrose gradient and centrifuged at 107,170× *g* for 2.5 h at 4 °C. The viral band at the 30%/60% interface was collected using an 18-gauge blunt cannula, resuspended in TSE buffer and subjected to 210,053× *g* centrifugation for 1.5 h at 4 °C. The pellet was collected and resuspended in TSE buffer to an appropriate minimal volume yielding between 10^8^–10^9^ PFU/mL.

### 2.2. Formalin Inactivation of Influenza Virus

The purified virus was inactivated with 10% formalin to a final concentration of 0.1% and incubated at 37 °C with constant nutation for 48 h. To test and confirm inactivation, an aliquot of the inactivated virus with PBS mock controls were diluted at 10^2^, 10^3^ and 10^4^ in 0.1% formalin. Virus inactivation was confirmed by the inability of the viruses to replicate in MDCK cells as observed by negligible CPE. The inactivated virus was stored at 4 °C.

### 2.3. Adjuvant and Vaccine Preparation

PCEP was synthesized by the Idaho National Laboratory (Idaho Falls, ID, USA) using methods previously described [9,16] and its endotoxin level was determined to be less than 0.034 ng/mL as assessed by the Limulus Amebocyte Lysate assay (Biowhittaker, Walkersville, MD, USA). Vaccines consisted of 4.0 × 10^4^ inactivated SIV HAU (haemagglutination units) alone or plus 4, 20, 100 or 500 μg PCEP; or 8.0 × 10^4^ inactivated SIV HAU alone or plus 4, 20, 100 or 500 μg PCEP. PBS was injected into control animals or animals were not immunized (naïve control animals). Inactivated SIV vaccines were diluted with PBS (pH of 7.4) into 250 μL per injection site. The highest dose of PCEP was chosen based on the previous experiments using PCEP in pigs [13,17,18].

### 2.4. Immunization with Inactivated H1N1 and Challenge with H3N2 Virus Experimental Design

All animal experiments were conducted in VIDO-InterVac (University of Saskatchewan, Saskatoon, SK, Canada) according to the Guidelines for the Care and Use of Laboratory Animals as indicated by the Canadian Council on Animal Care and was approved by the Animal Care Committee of the University of Saskatchewan (The Animal Use Protocol is AUP20170049).

Commercial cross bred pigs (3–4 weeks of age) were selected from sows prescreened 5 days before farrowing for negligible H1N1 and H3N2 antibody titers. The piglets were divided into 10 test groups (*n* = 6 in each group). Pre-existing H1N1 and H3N2 antibodies and H1N1 and H3N2 neutralizing antibody levels were quantified on all piglets prior to vaccination as marked by Day 0).

Please note that four piglets across several groups succumbed to *Streptococcus suis* infection within the acclimation period prior to start of the experiment and they were removed from data analysis. Necropsy was performed on these pigs with *S. suis* septicaemia determination. An independent analysis by Prairie Diagnostic Services, Inc. showed no detectable pneumonia or pleural cavity abnormalities. Two other pigs died by needle venous severance when the pig suddenly and forcibly moved while extracting blood during serum collection, and another pig died from umbilical hernia. Upon necropsy of these 3 animals, no detectable pneumonia or pleural cavity abnormalities were present. All remaining pigs were administered 1.3 mL of 100 mg/mL Baytril^®^ 100 (Bayer, Mississauga, ON, Canada).

**Figure 1:**
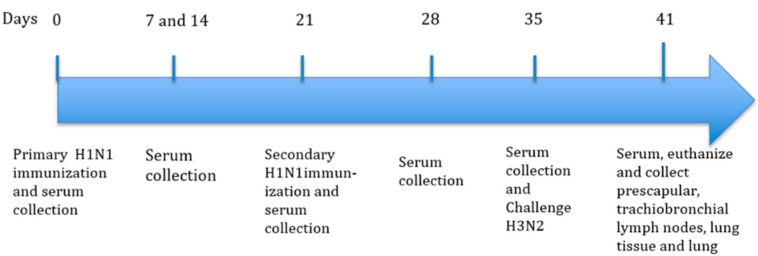
Schematic presentation of experimental design. Piglets were immunized via the intradermal injection with swine influenza virus (SIV) plus PCEP as an adjuvant, on day 0, and a secondary immunization was administered on day 21. Serum was collected for serum antibody titres assays. Clinical and injection site markings and scoring were performed up to day 41 after initial vaccination.

Pigs were immunized intradermally at the neck on day 0 (left side) and a booster vaccination given at day 21 (right side). The body temperature, clinical observations and score for local reactions at both injection sites were taken throughout the study period. The local reaction scores were from 0–3 with 0 = normal, 1 = minor, 2 = moderate and 3 = severe. No local reaction scores were observed (data not shown) for any vaccine formulation at any time point as described in Figure 1.

**Figure 2:**
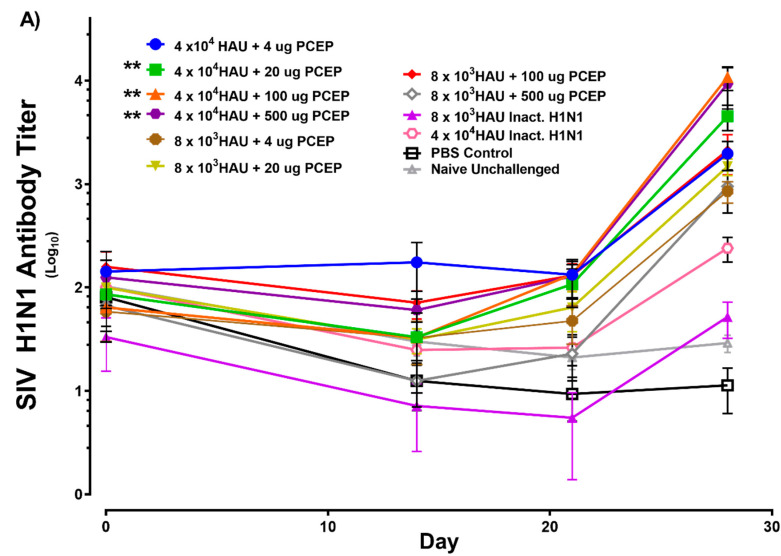

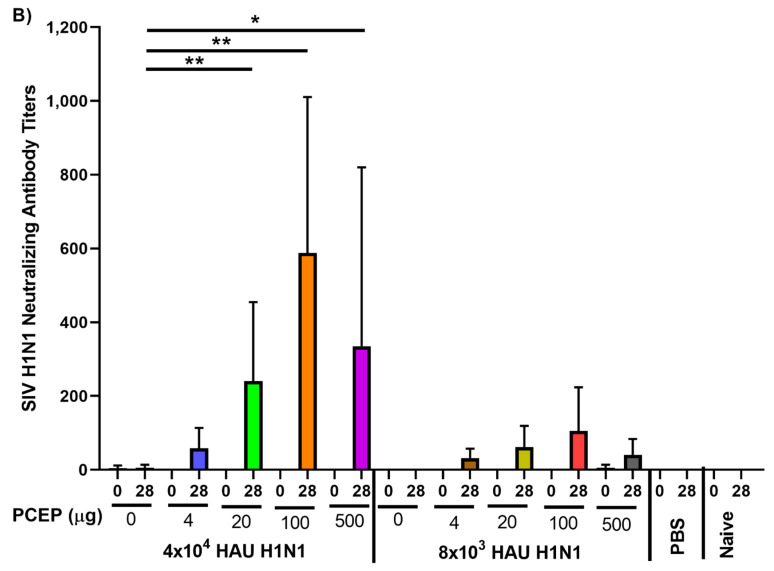
Vaccine-induced H1N1 antibody responses and neutralizing antibody responses in pigs. Pigs (*n* = 6 per group) were immunized with 4.0 × 10^4^ or 8.0 × 10^3^ inactivated SIV HAU alone or plus 4, 20, 100 or 500 μg PCEP. Control animals were either injected with PBS or not immunized (naïve). A secondary immunization was performed on day 21 and sera samples were collected over a period of 28 days. (**A**) Anti-H1N1 IgG antibodies were assessed by ELISA and (**B**) anti-H1N1 IgG neutralizing antibodies were assessed. Data for animal group are presented with mean values indicated by a horizontal bar and standard error of the mean are shown. *p* < 0.05 (*), and *p* < 0.01 (**).

Sera from each pig was collected 0, 14, 21, and 28 days post-vaccination and the antigen-specific antibody response (Figure 2A) and antibody neutralization effect against SIV H1N1 and H3N2 (Figure 2B) were assessed to select the groups with optimal antibody responses to the challenge.

**Figure 3:**
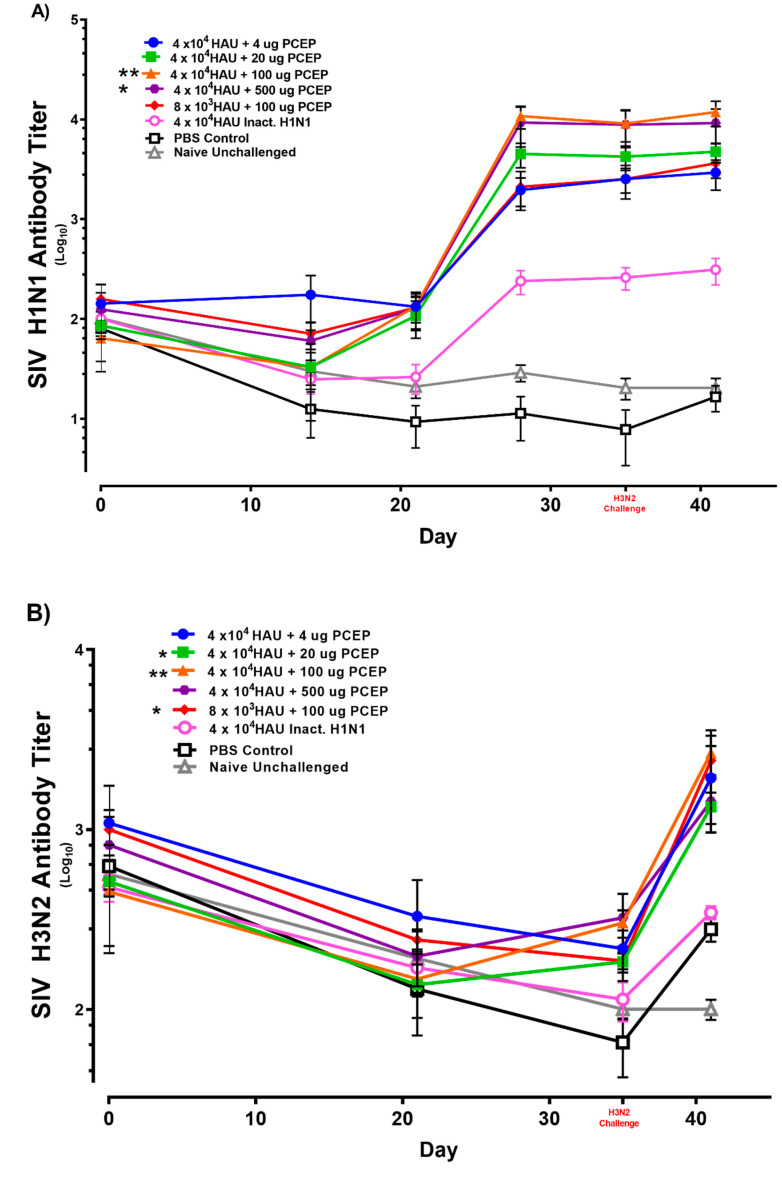

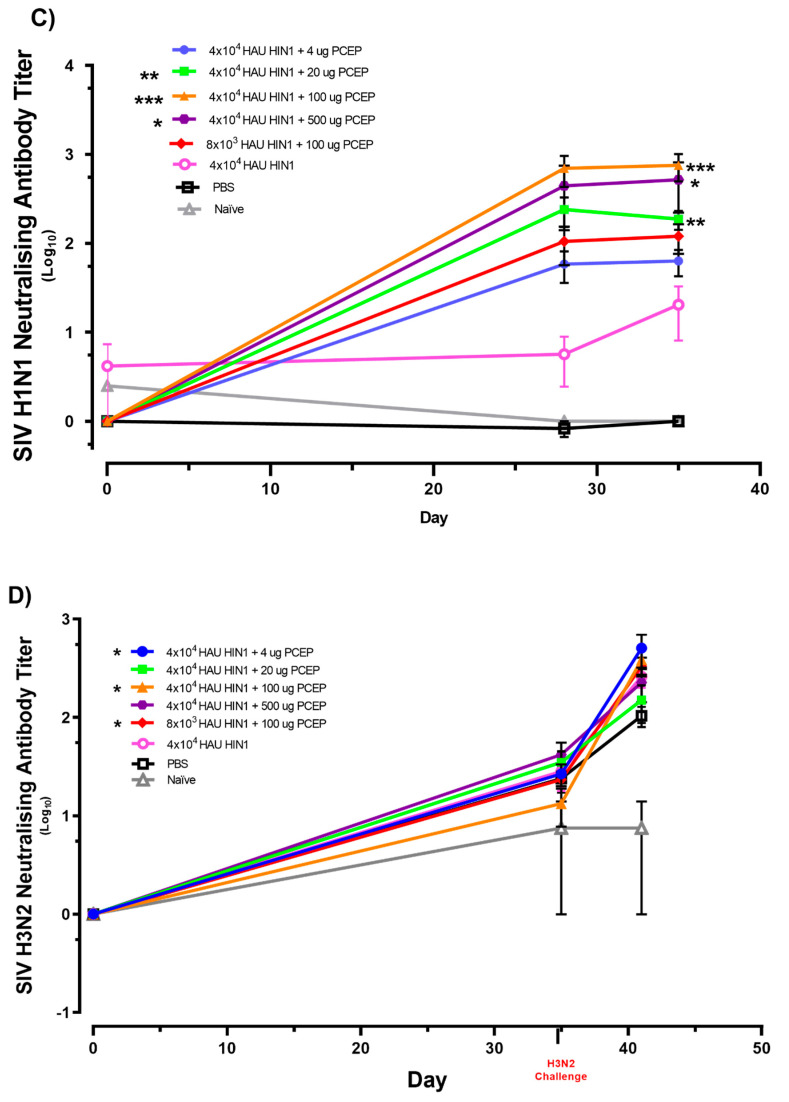
Swine influenza H1N1 and H3N2 ELISA and neutralizing antibody responses in pigs. Pigs (*n* = 6) were immunized then boosted at day 21 via intradermal routes with 4 × 10^4^ or 8 × 10^3^ HAU H1N1 either alone or with varying doses (4, 20, 100 or 500 μg) of PCEP adjuvant as indicated. Control groups were injected with either PBS or were unimmunized (naïve). They were challenged with 8 × 10^5^ PFU virulent H3N2 SIV via the intratracheal route on day 35 and killed 6 days after challenge. Serum antibody titres for SIV H1N1 (**A**) and H3N2 (**B**) were assayed by ELISA over time. Red font is used for the day of challenge for emphasis. Serum SIV H1N1 (**C**) and H3N2 (**D**) neutralizing antibody titres were assessed up to day 41. Data for animal groups are presented with mean values indicated by a horizontal bar and standard error of the mean are shown. *p* < 0.05 (*), *p* < 0.01 (**) and *p* < 0.001 (***).

Pigs were challenged with 8 × 10^5^ PFU virulent SIV H3N2 via the intratracheal route on day 35 using Strain A/swine/Texas/4199-2/1998/H3N2 (Tx98) as previously described by [19]. Challenged and naïve pigs were kept in separate rooms with no direct or indirect contract. Antigen-specific antibody response (Figure 3A,B) and antibody neutralization effect against SIV H1N1 and H3N2 (Figure 3C,D) were assessed up to day 41 post vaccination. All pigs in all groups were euthanized 6-days post-infection and samples were collected.

### 2.5. Necropsy and Macroscopic Examination of the Lungs

Animals in all groups were euthanized by intravenous administration of Euthanyl (25 mg sodium pentobarbital). For all animals, whole lungs were removed at necropsy and evaluated to determine the percentage of the lungs affected with purple-red, firm lesions that are typical of swine influenza virus infection [20]. The percentage of areas affected with pneumonia was estimated visually for each lung lobe. The total percentage lesions for the entire lung was calculated based on weight proportions of each lung lobe to the total lung volume as described by [21].

### 2.6. Detection of Swine Influenza Virus H1N1 and H3N2 Antibodies in Porcine Serum by Enzyme-Linked Immunosorbent Assay (ELISA)

Purified SIV H1N1 and H3N2 were inactivated by mixing one-part virus with one part 5% N-lauroyl sarcosine sodium salt (Sigma-Aldrich# L5125, Oakville, ON, CAN) and 8 parts PBS + 0.1% sodium azide and leaving at room temperature for 30 min. The viruses were diluted to 0.5 μg/mL and 1 µg/mL in 0.5 M bicarbonate buffer (coating buffer), respectively. Diluted virus was applied to separate Immulon 2 96U plates (Thermo Lab systems #3655) at 100 μL per well and the plates were incubated at 4 °C overnight. Experimental serum samples, and positive and negative serum control standards were diluted in PBS supplemented with 0.05% Tween-20 (Sigma-Aldrich Canada Co., Oakville, ON, Canada), transferred into coated-blocked plates, and 4-fold serially diluted. Antigen-specific total IgG were detected with alkaline phosphatase-conjugated KPL goat anti-swine IgG (H + L) (KPL, Gaithersburg, MD, USA), developed in PNPP substrate (1 mg p-nitrophenyl phosphate per mL in 1% diethanolamine with 0.5 mM MgCl_2_, pH 9.8) for 2 h, read at λ405 nm with a reference λ490 nm and the final titers were calculated.

### 2.7. Enzyme-Linked Immunosorbent SPOT Assay (ELISPOT) for IFN-γ, IL-13 and IL-17A

Tracheobronchial and prescapular lymph nodes draining the vaccination and challenge sites were dissected 6-days post-challenge and the cells were isolated and processed to generate a cell suspension following the protocol detailed in [22] with few modifications. The single-cell suspensions were restimulated with inactivated SIV H1N1 at the concentration of 25 μg/mL. The frequency of IFN-γ, IL-13 and IL-17A-producing cells was determined by ELISPOT. Under sterile condition, separate Millipore MultiScreen HA ELISPOT plates (Fisher Scientific # MAHAS4510, Thermo Fisher Scientific, Waltham, MA, USA) were coated overnight at 4 °C with Rabbit Anti-Porcine-IL-17A (Cedarlane# KP0498S-1000 (KingFisher Biotech Inc., St. Paul, MN, USA)), mouse Mab anti-porcine IFN-γ (ThermoFisher-EN-MP700, Thermo Fisher Scientific, Waltham, MA, USA) and Goat Anti-Po-IL-13 antibody (Cedarlane# PB0094S-100 (KingFisher Biotech Inc.)) diluted in coating buffer to 2, 5 and 3.5 μg/mL respectively. Plates were then washed four times with phosphate-buffered saline supplemented with 0.05% Tween 20 (PBST; Sigma-Aldrich) then 5 × 10^5^ cells were seeded in 100 μL AIM-V supplemented with 2% FBS medium per well in triplicate wells. Plates were incubated at 37 °C, 5% CO_2_, for 16 h (IFN-γ) or 35 h (IL-17A & IL-13), washed 5 times with PBST, and respective plates were incubated for 2 h with detection antibodies at either 0.2 μg/well biotinylated rabbit anti-pig-IL-17A (Cedarlane# KPB0499S-050 (KingFisher Biotech Inc.)), rabbit anti-pig IFN-γ (Fisher# # PP700) or 1 μg/mL Goat anti -Pig-IL-13 (Cedarlane# PBB0096S-050 (KingFisher Biotech Inc.)). IL-17A and IL-13 plates were washed 5× with PBST, and 100 μL of streptavidin-alkaline phosphatase was added and incubated for 90 min. After washing 8× in distilled water, 100 μL freshly prepared BCIP/NBT (Sigma-Aldrich# B5655, Oakville, ON, Canada) was added to each well and incubated for 3–20 min. Plates were washed with distilled water to stop the reaction and air-dried overnight before spot enumeration on an AID ELISPOT Reader model ELRO7 (IFL; AID GmbH, Strassberg, Germany) in conjunction with AID EliSpot Reader software version 7.0. The IFN-γ plates were treated with a secondary antibody, goat anti Rabbit IgG (H + L) biotin, for 2 h, prior to the 90 min 100 μL of streptavidin-alkaline phosphatase incubation, and BCIP/NBT developed similarly to the IL-17A and IL-13 plates. The number of IFN-γ and IL-17A and IL-13-producing cells per million cells from each tissue were determined by multiplying the number of spots per well by 2 and then averaging values from triplicate wells.

### 2.8. Swine Influenza Virus Neutralization Assay

Swine influenza virus neutralization was tested on serum samples that had been heat-inactivated at 56 °C for 30 min. Sera was tested in 4 replicates per serum dilution. Heat-inactivated serum samples were 10-fold diluted in unsupplemented MEM + 1 μg/mL TPCK-trypsin to a final volume of 60 µL in round bottom 96-well microtiter plates. The test virus was added (i.e., 60 µL) to all wells of the microtiter plate at 100 TCID_50_/50 μL swine influenza virus per well except the negative control. The virus-serum mixtures were gently agitated and incubated for 2 h at 37 °C in 5% CO_2_. Afterwards 100 µL of the virus-serum mixture was placed upon 90% confluent MDCK cells grown on tissue culture treated 96-well microtiter plates and incubated for 2 h at 37 °C in 5% CO_2_. Inoculum was removed, fresh MEM medium containing 1 μg/mL TPCK-trypsin and 50 μg/mL gentamicin was added and plates were incubated at 37 °C in 5% CO_2_ for 2 to 3 days. A corresponding Back Titration plate of 100 TCID_50_/50 μL at ½ log_10_ dilutions to 10^−2^ (i.e., 0.01 TCID_50_) was set up for each test virus and were subjected to the same incubation conditions as the serum-virus sample plates. Sample plates were scored when the back-titration plate for its corresponding virus showed 50% of the wells showing CPE at 1 TCID_50_.

### 2.9. Statistical Analysis

Statistical analyses were assessed using Graph-Pad Prism 7.03 software (GraphPad Software, San Diego, CA, USA). Analysis of gross lesions on lungs, antibody titres, ELISA titres and antibody neutralization between different treatment groups were compared using non-parametric Kruskal–Wallis test where Dunn’s multiple comparisons test was used post-hoc to identify statistically significant differences between different adjuvant test groups. *p* < 0.05 was considered to be statistically significant.

## 3. Results

### 3.1. Immune Responses after Intradermal Vaccination of Pigs with PCEP-Adjuvanted Inactivated SIV H1N1 Swine Influenza Virus Vaccine and Viral Protection Study

Pigs were injected intradermally with PBS (negative control) or with inactivated SIV H1N1 (4.0 × 10^4^ or 8.0 × 10^3^ HAU) alone or adjuvanted with 4, 20, 100 or 500 μg PCEP on day 0. A secondary immunization was administered 21 days later. Serum samples were collected over a period of up to 28 days and antigen-specific serum antibody titres were assayed by ELISA. Clinical analysis showed that there was no significant different in temperature nor did the pigs show any signs of coughing (data not shown). Serum collected from each piglet at day 0 show that pre-existing H1N1 and H3N2 antibodies were negligible prior to vaccination (Figure 2A). Further, negligible antibody titres were detected in piglets from all groups after the primary immunization suggesting that the primary response was not very robust (Figure 2A). After 28 days (7 days post-second immunization), pigs immunized with 4.0 × 10^4^ inactivated SIV HAU + 20 μg PCEP, 4.0 × 10^4^ inactivated SIV HAU + 100 μg PCEP, and 4.0 × 10^4^ inactivated SIV HAU + 500 μg PCEP showed significantly higher serum H1N1 antibody titres relative to the negative controls animals (Figure 2A; *p* < 0.01 for all), but not H3N2 antibody titres (data not shown).

Pre-existing H1N1 and H3N2 neutralizing antibody testing was quantified on all piglets prior to vaccination as marked by Day 0 (Figure 2B) and they were negligible. Piglets immunized with 4.0 × 10^4^ inactivated SIV HAU + 20 μg PCEP (*p* < 0.01), 4.0 × 10^4^ inactivated SIV HAU + 100 μg PCEP (*p* < 0.01) and 4.0 × 10^4^ inactivated SIV HAU + 500 μg PCEP (*p* < 0.05) showed significantly higher neutralizing anti-H1N1 serum antibody titres but not anti-H3N2 neutralizing antibody titres (Figure 2B) in serum after 28 days, which correlated well with the antibody response assayed by ELISA (Figure 2A) compared to naïve pigs (Figure 2B). Vaccines formulated with 8.0 × 10^3^ inactivated SIV HAU alone or plus PCEP failed to promote significant antibody and neutralizing antibody titres relative to the PBS controls and thus we selected 4.0 × 10^4^ HAU Inactivated SIV H1N1 as our vaccine dose for subsequent studies.

### 3.2. Immune Responses after Vaccination with Inactivated SIV H1N1 Swine Influenza Virus with PCEP Adjuvant after Challenge with Live H3N2

Next, we followed up piglets in the PCEP-adjuvanted 4.0 × 10^4^ HAU inactivated SIV H1N1 vaccinated groups with a challenge study using heterologous live SIV H3N2 to determine if piglets immunized against inactivated H1N1 were protected against virulent H3N2. Results show that 4.0 × 10^4^ inactivated SIV HAU + 100 μg PCEP and 4.0 × 10^4^ inactivated SIV HAU + 500 μg PCEP induced significant antibody responses against H1N1 (*p* < 0.05) and (*p* < 0.01), respectively) relative to the naïve unchallenged group at day 41 (Figure 3A). These antibodies were neutralizing against HIN1 in the groups immunized with 4.0 × 10^4^ inactivated SIV HAU + 20 μg PCEP, 4.0 × 10^4^ inactivated SIV HAU + 100 μg PCEP, 4.0 × 10^4^ inactivated SIV HAU + 500 μg PCEP doses (*p* < 0.01, *p* < 0.001, and *p* < 0.05, respectively) compared to naïve unchallenged group (Figure 3C).

When we assessed the H3N2 antibodies at day 41 (six days after challenge with H3N2), the groups vaccinated with 4.0 × 10^4^ inactivated SIV HAU + 20 μg PCEP, 4.0 × 10^4^ inactivated SIV HAU + 100 μg PCEP and 4.0 × 10^4^ Inactivated SIV HAU + 500 μg PCEP showed significantly induced antibody responses against H3N2 (*p* < 0.01), *p* < 0.01 and *p* < 0.05, respectively) at day 41 relative to naïve unchallenged group (Figure 3B). Further, groups vaccinated with 4.0 × 10^4^ inactivated SIV HAU + 4 μg PCEP, 4.0 × 10^4^ inactivated SIV HAU + 100 μg PCEP and 8.0 × 10^4^ inactivated SIV HAU + 100 μg PCEP (*p* < 0.05, *p* < 0.05 and *p* < 0.05, respectively) had significantly higher neutralizing antibody titers against H3N2 relative to naïve unchallenged group (Figure 3D) which can be attributed to the challenge with virulent H3N2 swine influenza virus six days earlier.

### 3.3. Cytokine Production from Lymph Nodes Collected from the Injection and Vaccination Site at Time of Termination

**Figure 4:**
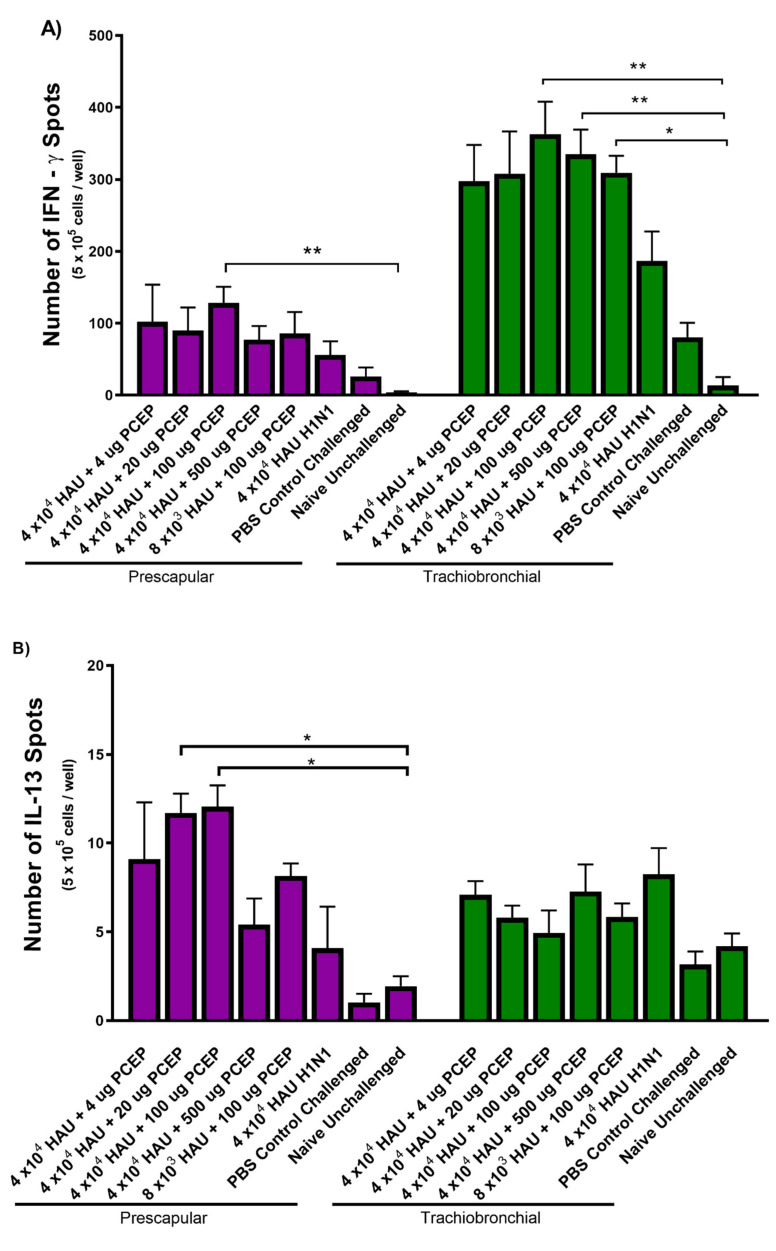

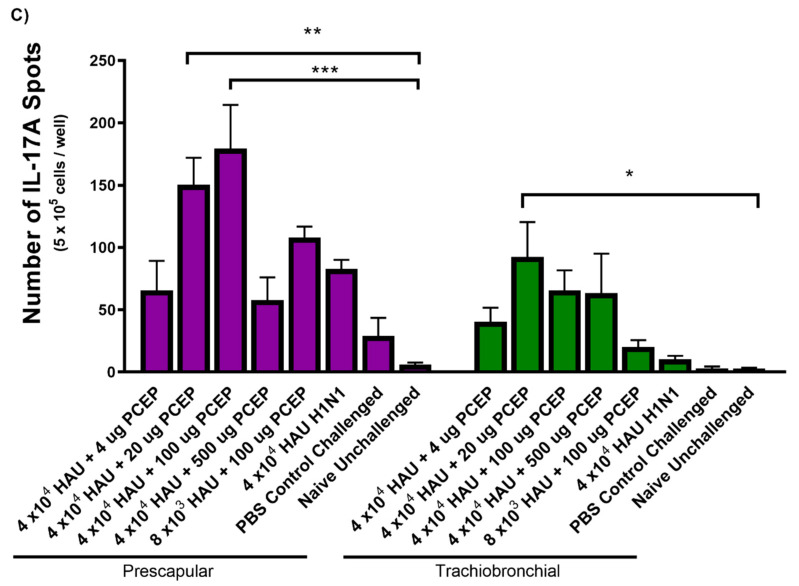
Adjuvant PCEP induces immune responses against inactivated swine influenza virus H1N1. Pigs (*n* = 6) were immunized then boosted at day 21 via intradermal routes with 4 × 10^4^ or 8 × 10^3^ HAU H1N1 plus 0–500 μg PCEP adjuvant as indicated. They were challenged with 8 × 10^5^ PFU virulent H3N2 SIV via the intratracheal route on day 35 and killed 6-days after challenge. Tracheobronchial and prescapular lymph nodes were collected and cells were harvested at 41 days post immunization. Individual single-cell suspensions of 5 × 10^5^ lymph node cells were re-stimulated with killed SIV H1N1 for 16 h for IFN-γ (**A**), and 48 h for IL-13 (**B**) and IL-17A (**C**) and measured by ELISPOT analysis. Data for animal groups are presented as a bar graph with the mean and standard error of the mean presented. *p* < 0.05 (*), *p* < 0.01 (**) and *p* < 0.001 (***).

At necropsy, we collected prescapular and tracheobronchial lymph nodes draining the vaccination and challenge sites, respectively. The lymph nodes were processed to single-cell suspension, and restimulated with inactivated SIV H1N1. We assayed for cytokine production by ELISPOT analysis to assess the antigen-specific cell-mediated immune responses. We observed significantly increased number of IFNγ-producing cells in tracheobronchial lymph nodes (i.e., the site of viral challenge) in animals immunized with 4.0 × 10^4^ inactivated SIV HAU + 20 μg PCEP, 4.0 × 10^4^ inactivated SIV HAU + 100 μg PCEP, 4.0 × 10^4^ inactivated SIV HAU + 500 μg PCEP and 8.0 × 10^3^ inactivated SIV HAU + 100 μg PCEP (*p* < 0.01, *p* < 0.01, *p* < 0.01 and *p* < 0.05, respectively) relative to the naïve unchallenged pigs. Relatively fewer IFNγ-producing cells were obtained from prescapular lymph nodes, the site of vaccine injection (Figure 4A). In contrast, pigs immunized with 4.0 × 10^4^ inactivated SIV HAU + 20 μg PCEP and 4.0 × 10^4^ inactivated SIV HAU + 100 μg PCEP showed significantly higher IL-13 secreting cells (*p* < 0.05 and *p* < 0.01, respectively) from the prescapular lymph nodes cells relative naïve unchallenged pigs. IL-13-secreting cells from tracheobronchial lymph nodes cells were not significantly increased in any vaccinated pigs relative to naïve unchallenged pigs (Figure 4B). Further, in pigs immunized with 4.0 × 10^4^ inactivated SIV HAU + 20 μg PCEP and 4.0 × 10^4^ inactivated SIV HAU + 100 μg PCEP, we observed significantly higher IL-17A secreting cells (*p* < 0.01 and *p* < 0.001, respectively) from cells from the prescapular lymph nodes cells relative to naïve unchallenged pigs (Figure 4C). IL-17A-secreting cells were not significantly increased in tracheobronchial lymph nodes of any vaccinated pigs relative to either control group (Figure 4C).

### 3.4. Lung Lesion Score

**Figure 5:**
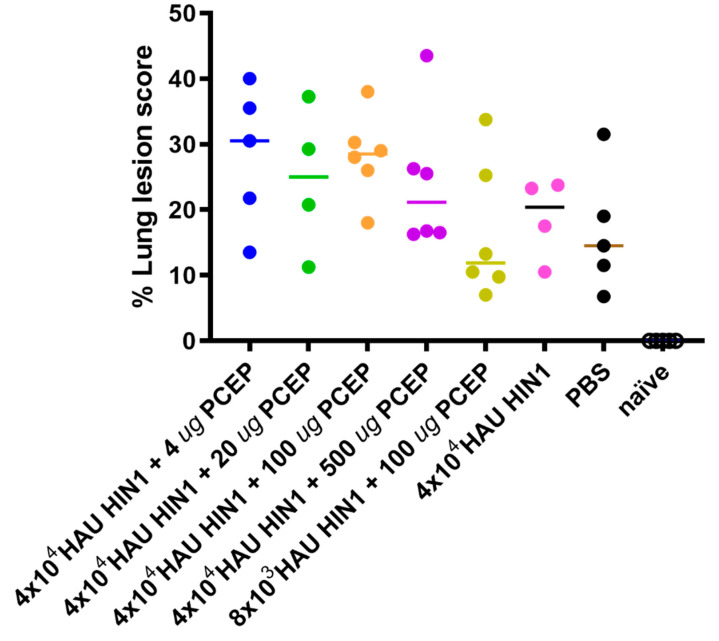
Lesion scores in lungs of vaccinated and challenged animals. Pigs (*n* = 6) were immunized then boosted at day 21 via intradermal routes with 4 × 10^4^ or 8 × 10^3^ HAU H1N1 plus 0–500 μg PCEP adjuvant or PBS as indicated. Naïve animals were neither immunized, nor challenged. The challenge dose was 8 × 10^5^ PFU virulent H3N2 SIV via the intratracheal route on day 35 and all animals were killed 6 days after challenge. The lung lesion scores were assessed (as indicated in the methods) up to 6-days post challenge. Each data point indicates an individual animal and the horizontal bar represents the median. The colors of each data point are coordinated with the group colors from Figure 1, Figure 2 and Figure 3.

Previously, we showed that pigs immunized intradermally, with inactivated H1N1 swine influenza virus were protected against homologous challenge [13]. Here, animals were challenged with heterologous SIV H3N2 virus to determine whether the PCEP-adjuvanted H1N1 vaccine was cross-protective. All challenged animals had lung lesions (Figure 5) indicating that the vaccine did not protect against heterologous challenge.

## 4. Discussion

Swine influenza is widely prevalent in swine herds in North America and Europe causing significant economic losses to the pig industry and it is a public health threat [23,24,25]. Pigs can be infected by both avian and mammalian influenza viruses and co-infection can lead to a reassortment of influenza viruses capable of causing pandemics in humans [26,27]. Should reassortment occur, the resultant influenza virus can be transmitted from person to person, and may cause more severe disease in humans than the original viruses [27]. For these reasons, vaccination of pigs would protect humans from future influenza outbreaks.

Vaccination is the most efficient method of protection against influenza infections [26,28] but the rapidly mutating virus has resulted in diversity of contemporary swine influenza virus strains causing an impediment in the development of new influenza vaccines [29]. Current commercial vaccines provide satisfactory immunity against homologous viruses but protection against heterologous viruses is not adequate [30]. Because of the high mutation rate of SIV and reassortment in pigs, each year’s circulating vaccine strains can vary widely and lead to the sudden emergence of substantially different strains which can trigger a human pandemic [31,32]. If this should happen, a vaccine shortage is likely to occur since traditionally used adjuvants, such as alum, have not shown significant antigen sparing and are not effective adjuvants for H5N1 and pandemic H1N1 influenza virus vaccines [33,34]. Hence, there is a need for a new generation of adjuvant capable of inducing protective immune response that induces cross protection against different strains of influenza viruses.

Some authors claim that mucosally delivered attenuated virus vaccines have the potential to provide broad cross-protection [35] which is attributed to the fact that infected cells recognize influenza virus RNA via pattern-recognition receptors (PRRs). Activation of PRRs stimulates production of pro-inflammatory cytokines and type I interferons [36]. Additionally, live-attenuated influenza vaccines have an advantage over inactivated products because they mimic a natural route of infection. Inactivated vaccines are poor inducers of innate immunity and they generally lead to immediate protection. In contrast, vaccination with live products provides both humoral and cell-mediated immunity, and they can induce mucosal IgA responses in the upper respiratory tract thus providing more comprehensive cross-reactive and longer-lasting immune responses [37,38].

However, despite their capacity for cross-protection, live vaccines do pose significant risks for reversion to virulence and disease, and also the potential for shedding which creates public health concerns. In contrast, inactivated vaccines a long track record of safety but they require formulation with adjuvants to improve their immunogenicity and protection and the potential for cross-protection against challenge with heterologous influenza strains. Clegg et al. reported that the combination adjuvant GLA-SE, but not the commercial SE adjuvant, protected against heterosubtypic H5N1 challenge in mice and ferrets [7]. This cross-protection was apparently mediated via induction of Th1-mediated antibody responses [7]. Thus, adjuvants that induce broad immune responses that include Th1- and Th2-type immune responses can potentially mediate cross-protection.

We have reported that the experimental adjuvant PCEP promotes strong antigen-specific Th1- and Th2-type immune responses to influenza antigens in mice and pigs [10,13,14]. Further, intradermal administration of an inactivated H1N1 SIV vaccine formulated with PCEP in pigs induced both systemic (both humoral and cell-mediated) and mucosal immune responses as indicated by reduced lung viral titres in pigs challenged with homologous H1N1 virus [13]. The broad-spectrum immune responses induced by PCEP-adjuvanted influenza vaccine suggested to us that this adjuvant can confer cross-protection. Thus, in the present study, we investigated the immunogenicity and protective potential of the same vaccine and assessed its protective efficacy against a tracheobronchial challenge with the heterologous H3N2 virus strain. The current vaccine induced strong SIV H1N1-and H3N2-specific systemic antibody and neutralizing antibody production. Further, lymph nodes draining the site of challenge (tracheobronchial) but not the site of H1N1 immunization, had significant induction of cells that secreted IFN-γ at the site of heterologous challenge which is consistent with our previous results [13]. The lymph nodes draining the site of injection had significant induction of cells that secreted IL-13 and Il-17A six days after the heterologous challenge when re-stimulated with SIV H1N1. Similar to our recent findings, other study shows that subcutaneous immunization in mice utilizing LPS as an adjuvant resulted in a CD4 T cell mixed response in the sense that cells capable of secreting IFN-γ, IL-4 or IL-17 were induced to varying degrees [39]. Effective influenza vaccination is currently assessed by anti-influenza antibody levels due to the accepted and established importance of humoral immunity for protection [40]. However, studies have shown that cellular responses play an important role in protection, with associations drawn between pre-existing elevated IFN-γ -producing influenza-specific CD4^+^ and CD8^+^ T-cells and less severe disease [41,42] which is probably the reason for increased IFN-γ production at the site of challenge. These results suggest that the vaccines induced humoral immunity as well as a mixed Th1/Th2 type T cell response. In agreement with our current findings is another study that established that immunization of piglets with live or attenuated swine influenza virus primed both the CD4^+^CD8^+^ and CD4^+^CD8^−^T-cell populations for early IFN-γ recall responses [35]. In our study, animals with high antibody and neutralizing antibody responses against H1N1 had high lung lesion score when challenged with H3N2 suggesting that no cross protection occurred and that over stimulation of immune system with one antigen may lead to a more severe infection with none cross protective strains. As observed in our studies, the level of lung lesion score after heterologous H3N2 challenge is higher in vaccinated pigs than unvaccinated pigs. However, non-neutralizing or low-affinity neutralizing antibodies following vaccination or infection have also been correlated with augmenting influenza disease post-infection in other animal models and several human observational studies [43,44]. Interestingly, in our study the H1N1-induced antibodies did not neutralize H3N2 after challenge nor did the H1N1-adjuvanted vaccine induce antibodies specific for H3N2 targets. Further, the percentage of lung lesion scores after heterologous H3N2 challenge was not significantly higher in vaccinated groups than unvaccinated (PBS injected) pigs (Figure 5). However, others studies have reported that the lack of neutralizing antibodies or low-affinity neutralizing antibodies following vaccination or infection correlates with augmenting influenza disease post infection or vaccination in other animal models [43,45,46]. This condition is referred to as antibody-dependent enhancement (ADE) of influenza disease and pathology [45]. The frequency and severity of ADE depends on the levels of vaccine-induced non-neutralizing antibodies in the lower respiratory tract, and their interaction with various innate cells via FcR and activation of complement [47].

In contrast to our results, others showed that vaccination of pigs intranasally with NS1-truncated H3N2 swine influenza virus primed T cells and conferred cross-protection against an H1N1 heterologous challenge [35]. Our studies show that polyphosphazene are potent immunostimulants in mice and pigs that enhance the magnitude of the immune response as well as alter the quality of immune response when administered intranasally and intradermally with influenza antigens [13,14]. However, as postmortem lung scores did not differ between vaccinated and control piglets, we must conclude that this intradermal vaccine formulation did not cross protect against experimental heterologous H3N2 challenge.

## 5. Conclusions

The adjuvant PCEP induced a variety of antigen-specific immune responses against H1N1 including, neutralizing antibodies, and IFN-γ, IL-13, IL-17A in the draining lymph nodes in pigs. These cellular and humoral antigen-specific immune response against H1N1 were protective against challenge with homologous virus (H1N1), but were not cross protective against the heterologous H3N2 virus.
